# N-acetylcysteine as an adjunct treatment of schizophrenia

**DOI:** 10.1192/j.eurpsy.2021.1418

**Published:** 2021-08-13

**Authors:** R. Mota Freitas, M.T. Valadas

**Affiliations:** 1 Departamento De Psiquiatria E Saúde Mental, Hospital do Espírito Santo de Évora, Évora, Portugal; 2 Serviço De Psiquiatria, Unidade Local de Saúde do Baixo Alentejo, Beja, Portugal

**Keywords:** schizophrénia, N-acetylcysteine

## Abstract

**Introduction:**

An increasing body of literature supports the hypothesis that immune imbalance towards a pro-inflammatory status in the brain plays an important role in schizophrenia. Anti-inflammatory drugs might compensate this dysregulation, ameliorating the symptoms of schizophrenia. N-acetylcysteine exhibits anti-inflammatory properties and may regulate various neurological pathways, including glutamate dysregulation, oxidative stress, and inflammation, becoming an interesting augmenting drug for schizophrenia treatment.

**Objectives:**

We aim to review the literature regarding the therapeutic effects of N-acetylcysteine in Schizophrenia.

**Methods:**

We performed an updated review in the PubMed database using the terms “N-acetylcysteine” and “Schizophrenia”. The included articles were selected by title and abstract.

**Results:**

The literature suggests that N-acetylcysteine may be a useful adjunct to standard treatment for the improvement of schizophrenia symptoms, as well as the cognitive domain of working memory. Also, this augmentation therapy seems to be beneficial in all illness stages

**Conclusions:**

N-acetylcysteine appears to be a promising agent for augmenting conventional pharmacotherapy in schizophrenia, however, further research is needed to consolidate the current findings.

